# Phenology and Abundance of Migratory and Breeding Populations of Monarch Butterflies in the Pacific Northwest 2012–2024

**DOI:** 10.3390/insects17010001

**Published:** 2025-12-19

**Authors:** David G. James, Tanya S. James

**Affiliations:** 1 Department of Entomology, Washington State University, Irrigated Agriculture Research and Extension Center, 24106 North Bunn Road, Prosser, WA 99350, USA; 2 Washington State Department of Agriculture, 21 North First Street, Yakima, WA 98902, USA; tanya.james@agr.wa.gov

**Keywords:** phenology, abundance, community science, sightings, fluctuations, climate

## Abstract

The decline of the monarch butterfly population in western North America has been documented by 28 years of community scientist counts from overwintering sites in California. However, no long-term studies on the status and dynamics of monarch populations during spring to fall are available. Using multiple datasets from online recording sites, social media, and personal observations, our study over 13 years shows increases and declines in warm-season populations in the Pacific Northwest (PNW) on a 2 to 4-year cycle that corresponds with fluctuations in overwintering populations. Overall, there was little evidence of a systemic decline in the abundance of monarchs in the PNW. Monarchs were reported in all PNW states and territories in all years, with the earliest annual first appearance in Oregon and the latest in British Columbia and a maximum seasonal range of 200 km north of the US/Canada border. Relatively equal numbers of unique sightings originated from each of three community scientist online recording platforms, with a larger number of unique sightings from personal observations/communications and multiple social media sites. The value of community scientist sightings for the rapid detection of substantial changes in populations was highlighted in August 2024 when weekly sighting data showed a substantial decline in PNW populations that may have been caused by poor survival of immature stages during a July heatwave. The conservation of monarchs in western North America will benefit from contemporary and high-value data on phenology and population dynamics, which we show can be provided by community science observations.

## Simple Summary

Reduced numbers of overwintering monarch butterflies in California indicate a decline in the species in western North America, but little attention has been given to the status of warm-season populations in the west. Using sighting data contributed largely by community scientists, we evaluated the phenology and abundance of monarchs during spring, summer, and fall in the Pacific Northwest (PNW) over 13 years. Periods of higher and lower abundance corresponded with overwintering numbers, confirming population linkages between the PNW and California. Sighting data showed a late-summer collapse of monarch populations in 2024, highlighting the value of real-time online reporting by community scientists. Effective strategies for the conservation of monarchs in western North America will benefit from high-value, current information on population dynamics, which we show can be provided by community science data.

## 1. Introduction

Monarch butterflies, *Danaus plexippus* L. (Lepidoptera: Nymphalidae), in western North America undergo a fall migration to the California coast, where overwintering occurs in aggregated colonies at more than 300 sites. Following colony break-up in February–March, butterflies migrate inland, producing a generation of immatures within California. Adults from this generation migrate north and east during April–June, colonizing the full western range of the species [1,2]. Populations of monarch butterflies in western North America have declined since the late 1990s, as assessed by annual counts of overwintering populations in coastal California [2,3]. The monarch is currently proposed to be listed as threatened under the Endangered Species Act in the United States [4]. The Western Monarch Thanksgiving Count has provided annual estimates of populations at more than 250 overwintering sites since 1997. Citizen monarch counters are trained to count roosting monarchs, and counts are considered to be reasonably accurate given that the numbers at sites range from hundreds to thousands and not the hundreds of thousands typical of Mexican overwintering sites. From a historical high of more than a million butterflies overwintering in California in the late twentieth century, numbers fell at the turn of the century to ~200,000–400,000 [1,2]. For 16 years (2001–2017), winter counts consistently ranged between 58,000 and 300,000 butterflies before another substantial decline to ~30,000 butterflies occurred in 2018. A three-year period of very low numbers (~2000–30,000) during 2018–2020 was followed by a rebound to ~234,000–335,000 during 2021–2023, then a decline to ~9000 in 2024 [2,3]. While we cannot be sure that non-breeding overwintering populations and their fluctuations accurately represent the status of the western North American monarch population (winter-breeding populations are increasingly present in Arizona and California [5]), they are, at minimum, a useful barometer of monarch populations in western North America. The source areas of monarchs migrating to California each fall comprise all the western US states and the Canadian province of British Columbia [6,7,8,9]. The Pacific Northwestern states of Oregon and Washington are a source area for monarchs migrating to California for overwintering, as shown in recent tagging research [10,11]. Most recovered monarchs that were tagged in Washington, and all of those that were tagged in Oregon, were found in California, often at overwintering sites. Only two Idaho-tagged monarchs were recovered in California [10,11], with most recoveries found up to 160 km to the south or south-east in Idaho, suggesting that the majority of Idaho monarchs do not migrate to California. However, the recovery of a northern California-tagged monarch in Idaho in spring confirms that some migration to and from Idaho does occur [9].

Data on the distribution and abundance of breeding populations of *D. plexippus* in the west are limited and mostly confined to short or discontinuous time frames. For example, James [12] studied summer-breeding populations at a single site in central Washington for three years (2013–15). Waterbury et al. [13] surveyed breeding populations in Washington and Idaho for two years (2016–17), and Morris et al. [14] provided data on monarch populations in Arizona over a 10-year period but not on an annual basis. There are no published long-term, large geographical scale annual studies on breeding populations that could be correlated with the long-term censusing of overwintering monarchs [3]. Overwintered monarchs are the source of subsequent year breeding populations in the west, the success of which, in turn, determines the size of subsequent overwintering colonies. Long-term information on the success and abundance of breeding populations of monarchs in the western US may help us better understand the annual fluctuations of overwintering colonies. Similarly, data on the phenology of migration and breeding in the summer range of monarchs in the west will improve our understanding of the seasonality and the annual population expansion and contraction of monarchs in this region. Our aim in this paper is to address these deficiencies by collating and presenting community science-derived observation data on monarch incidence and abundance during spring–fall in the Pacific Northwest. An additional objective was to provide data to confirm our hypothesis of a linkage between overwintering colonies in California and warm-season populations in the Pacific Northwest.

In this study, datasets of verified monarch sightings from three citizen science platforms, an annual research study at a migration corridor site, and sightings derived from social media/personal communications and observations were combined to show the annual phenology and abundance of migratory (spring and fall) and non-migratory (summer) monarch butterflies in the Pacific Northwest over a 13-year period (2012–24). These data broadly accord with trends shown by overwintering colonies in California and show the influence of spring to fall breeding on subsequent winter colonies.

## 2. Materials and Methods

Data on sightings of monarch butterfly adults and immature stages (eggs, larvae, and pupae) during 2012–24 in Oregon (OR), Washington (WA), Idaho (ID), and British Columbia (BC) were obtained from three online community science platforms, personal communications, and online social media. The community science platforms were iNaturalist (http://inaturalist.org accessed on 31 March 2025), Journey North (http://journeynorth.org accessed on 31 March 2025), and Western Monarch Milkweed Mapper (http://monarchmilkweedmapper.org accessed on 31 March 2025 OR, WA, and ID only). Personal communications and online social media comprised a database of personal observations, emails, and reports from social media, primarily Facebook (http://facebook.com) pages devoted to Pacific Northwest (PNW) monarch butterflies, butterflies and moths, or insects (e.g., Facebook pages such as Monarch Butterflies of the Pacific Northwest, Idaho Friends of Monarchs, Southern Oregon Monarch Advocates, Western Monarch Advocates, Butterflies and Moths of the Pacific Northwest, Eastern Washington/North Idaho Insects and Spiders, and Pacific Northwest Entomology). Only sightings of monarchs on social media that were verified either by photograph or sighter credentials (i.e., identified by an acknowledged expert) were included in our database. Some other butterflies (e.g., Tiger Swallowtails (*Papilio* spp.), Viceroy (*Limenitis archippus* (Cramer)), and California Tortoiseshell (*Nymphalis californica* (Boisduval)) are sometimes misidentified as monarchs. All monarch sighting data on the community science platforms were reviewed and verified by DGJ before inclusion in our database. Online social media sites were reviewed daily during spring–fall, and monarch sighting data was extracted. Personal communications comprised personal (DGJ) sightings recorded in field notebooks during summer fieldwork, and emailed records of sightings (with images) were sent to DGJ during 2012–24. Although less formalized than online reporting sites, this dataset, along with informal reporting on social media platforms, produced a large number of unique and verifiable sightings. Duplicate sightings reported on more than one platform or source were culled to ensure all recorded sightings were unique. Sighting data reported in the Western Monarch Milkweed Mapper that were associated with professional surveys were excluded to avoid bias. Similarly, survey data previously reported for Lower Crab Creek, WA [12], were also excluded. Some genuine data points for monarchs were likely excluded because of lack of verification. Sighting data were collected and categorized as spring (1 April–30 June), summer (1 July–31 August), or fall (1 September–31 October) sightings for each year. Most spring sightings before 1 July and fall sightings after 1 September were of northerly and southerly migrants, respectively. All sightings in July and most sightings in August were of breeding individuals. Data were sorted by state or province before combination to provide an overall PNW picture. In the final three years of the study (2022–24), sighting data from 1 June–31 October were also compared on a weekly basis to better define seasonality and variation in phenology and abundance between the three years. Analysis of Variance (ANOVA) was used to compare the number of monarch sightings between the different reporting datasets used and also to compare the number of sightings between states and provinces. Raw data were log (log x) transformed prior to analysis to improve normality of variances and then back-transformed for reporting. Means were separated using the Holm–Sidak method for comparing multiple groups (SigmaStat Version 3.0. SPSS Inc., Chicago, IL, USA).

Data on occurrence and numbers of monarch butterfly adults and immature stages were also obtained during annual field trips (2012–24) to a monarch migration corridor/breeding site along a 15 km section of the Trinity River in northern California (92 km south of the CA/OR state line), centered near Eagle Creek (41°09′ 03.97 N 122°40′ 15.03 W). Surveys at this site were conducted each year on the Memorial Day long weekend in late May, coinciding with the beginning of significant monarch migration into the PNW. For each survey, eight hours were spent during one or two days searching for adult monarchs at five or six sites spanning the 15 km corridor. Milkweed (*Asclepias speciosa* Torr. and *Asclepias fascicularis* Decne.) stems at each site were examined for the presence of eggs and larvae and counted. Searches were conducted under sunny, warm (20–35 °C) conditions (10 a.m.–4 p.m.).

## 3. Results

### 3.1. Abundance of Monarchs During Late May 2012–24 Along the Trinity River, Northern CA

**Figure 1:**
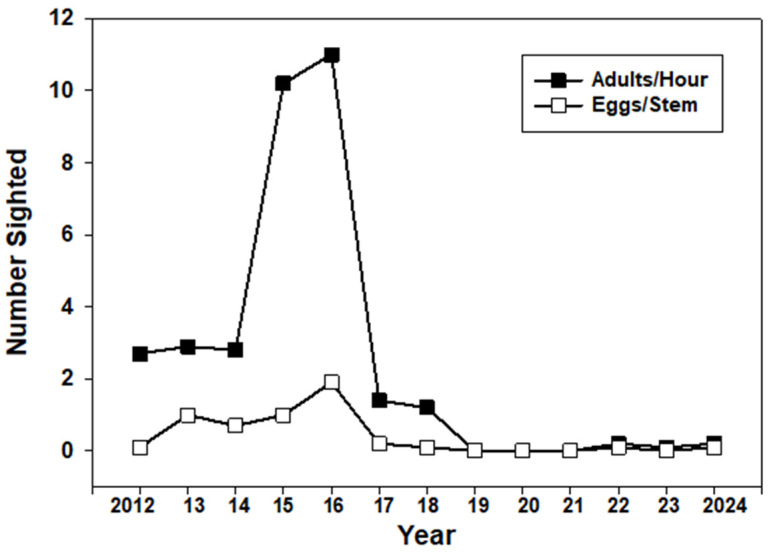
Monarch adults and eggs recorded during annual visits to a 15 km section of the Trinity River in Northern California on the Memorial Day long weekend during 2012–24.

The abundance of monarchs along the Trinity River during Memorial Day long weekends varied substantially during the 13 years of observation (Figure 1). During 2012–14, populations were similar, with 19–31 adults seen over two days (1.6–2.6 per hour), and examination of 100–200 milkweed stems yielded means of 0.03–0.2 eggs and early instar larvae/stems. Observations indicated that the population each year comprised migrants in good to fresh-wing condition heading north along the river together with wing-worn individuals that appeared to be residential near patches of milkweed, with females ovipositing and males showing territorial behavior. The numbers of butterflies increased substantially in 2015 and 2016, with 102 and 110 adults sighted (8.5–9.2/h) and means of 1.5 and 1.9 eggs/stem (50–100 stems). There were also significant numbers of early instar larvae. During these years, migrants flew north along the river at a rate of 2–3 every 5 min, sometimes into a northerly headwind. In 2015, a sample of 21 monarchs flying along the river was tagged, and 1 was recovered five weeks later 707 km ENE in Twin Falls, ID [10]. The sex ratio was heavily skewed towards males (75% in 2015, 95% in 2016). The abundance of monarchs along the Trinity River in late May 2015 and 2016 resulted in a small number of individuals killed by motor vehicles traveling on US Hwy 3 parallel to the river. In 2016, a dozen migrating monarchs were observed forming a roost in late afternoon in a single pine tree near the river. In 2017 and 2018, monarch numbers dipped back to 2012–14 levels (1.0–1.2/h). Monarchs were absent from the site in 2019, 2020, and 2021. Small numbers (0.2/h) were present along the river during 2022–24 (Figure 1).

### 3.2. Annual First and Last Sightings of Monarchs in the PNW 2012–24

Mean dates of first sightings of monarch eggs, larvae, and adults in BC occurred later in the season (22 June–10 July) than in WA (5–30 June), ID (1–17 June), and OR (11 May–12 June) (Table 1). The first adult sighting in each state varied by ~two months and was as early as 10 April in OR and as late as 29 July in BC. Similarly, there was much variation in the mean last-sighting dates for adults ranging from early August in BC to mid-October in OR. A trend towards later final sightings resulted in exceptionally late dates for OR (8 November), WA (28 October), ID (25 October), and BC (8 October), all since 2019 (Table 1).

### 3.3. Monarch Spring Sightings in the PNW (2012–24)

**Figure 2:**
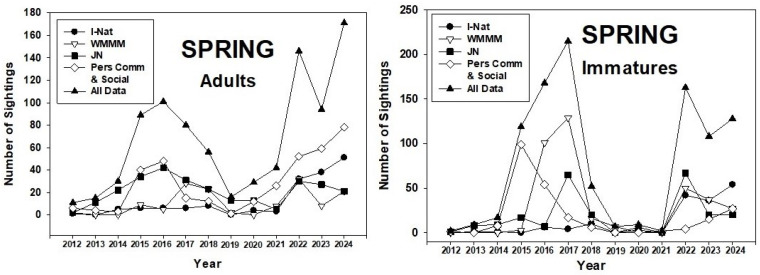
Spring (1 April–30 June) adult and immature monarch sightings in the Pacific Northwest during 2012–24. Sighting data obtained from inaturalist.org, westernmonarchmilkweedmapper.org, journeynorth.org, and from social media and personal communications.

The number of spring sightings of adult monarchs in the PNW ranged from 11 (2012) to 171 (2024). There were two three-year peaks in sightings (2015–2017, 2022–2024), with low numbers prior to and between the peak years (Figure 2). Sightings of immatures followed the trend shown by adults.

### 3.4. Monarch Summer Sightings in the PNW (2012–24)

**Figure 3:**
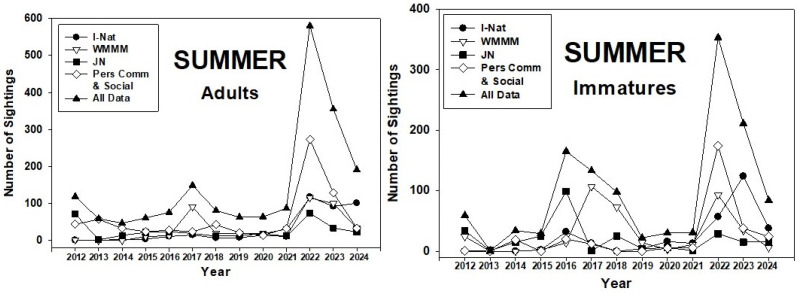
Summer (1 July–31 August) adult and immature monarch sightings in the Pacific Northwest during 2012–24. Sighting data obtained from inaturalist.org, westernmonarchmilkweedmapper.org, journeynorth.org, and from social media and personal communications.

The number of summer sightings of adult monarchs in the PNW ranged from 47 (2014) to 580 (2022) (Figure 3). While there were small peaks in numbers in 2012 and 2017–2018, the greatest numbers were seen during the summers of 2022–24. Sightings of immatures followed the same trend as adults.

### 3.5. Monarch Fall Sightings in the PNW (2012–24)

**Figure 4:**
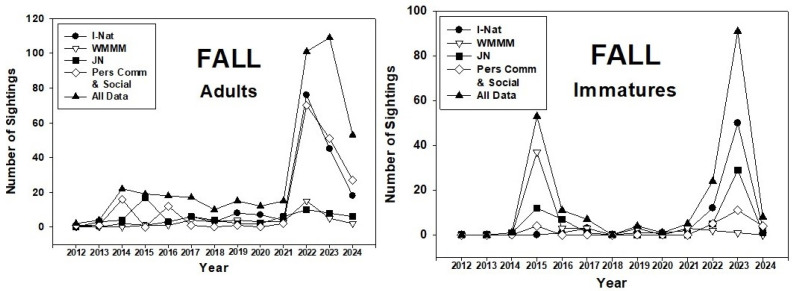
Fall (1 September–31 October) adult and immature monarch sightings in the Pacific Northwest during 2012–24. Sighting data obtained from inaturalist.org, westernmonarchmilkweedmapper.org, journeynorth.org, and from social media and personal communications.

The number of fall sightings of adult monarchs in the PNW ranged from 2 (2012) to 109 (2023) (Figure 4). Numbers were consistently low (10–20) until 2022–23, when more than 100 were reported in both years. Sightings of immatures followed the same trend as adults apart from a spike in 2015.

### 3.6. Monarch Spring to Fall Sightings in the PNW (2012–24)

**Figure 5:**
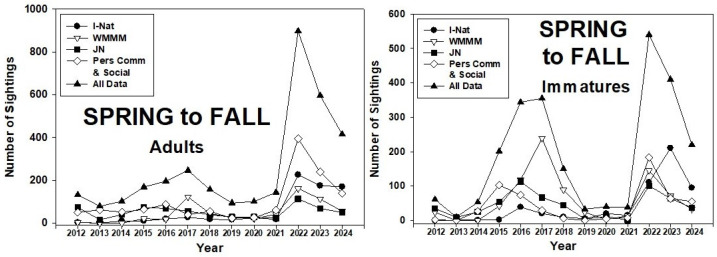
Season-long (1 April–31 October) adult and immature monarch sightings in the Pacific Northwest during 2012–24. Sighting data obtained from inaturalist.org, westernmonarchmilkweedmapper.org, journeynorth.org, and from social media and personal communications.

The number of adult monarch sightings each spring to fall season in the PNW ranged from 79 (2013) to 897 (2022) (Figure 5). There were two three-year peaks in sightings (2015–2017, 2022–2024), with low numbers prior to and between the peak years. Sightings of immatures followed the trend shown by adults, with a more distinct peak during 2015–17.

### 3.7. Weekly PNW Adult Monarch Sightings 1 June–31 October 2022–24

**Figure 6:**
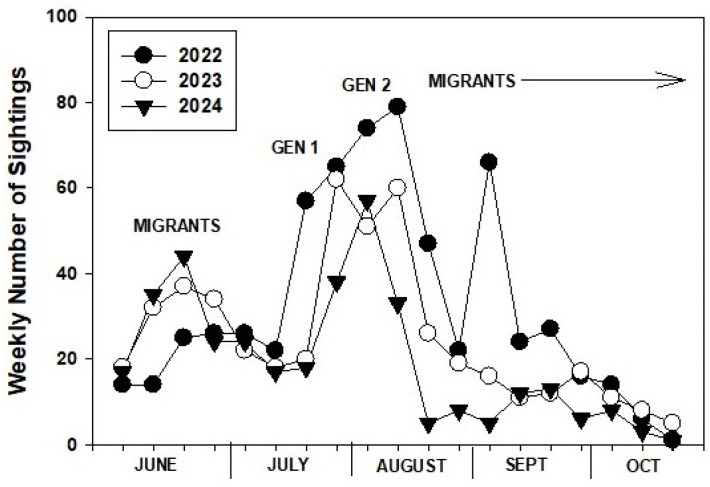
Weekly sightings of monarch adults in the PNW during 1 June–31 October 2022–24. Sighting data obtained from inaturalist.org, westernmonarchmilkweedmapper.org, journeynorth.org, and from social media and personal communications.

Weekly sightings of adult monarchs during summer and fall in the PNW over the final three years of the study showed commonalities in phenology and abundance (Figure 6). An early peak in sightings during mid–late June represented the incoming migrant population followed by a decline as these butterflies died off. The first PNW-produced generation of adults in mid–late July was strong in 2022 and 2023 but was later and weaker in 2024. The second generation of adults occurred during August and was strong in 2022 and 2023 but weak in 2024. This was most notable in the second half of August 2024, with 5–8 sightings compared to 20–47 sightings in 2022 and 2023 (Figure 6). Sightings in 2022 and 2023 declined in the third week of August as migrants headed south, but the drop in 2024 occurred in the second week, prior to major migration.

### 3.8. Comparison of Source Influence on Monarch Sightings

**Figure 7:**
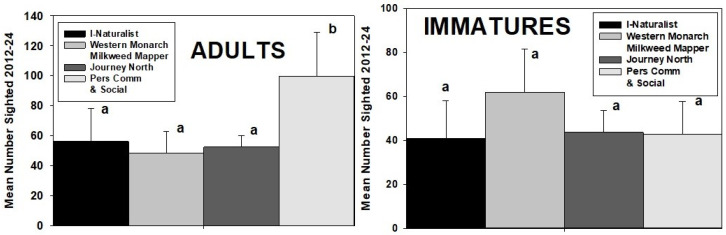
Mean (±SE) number of unique annual adult and immature monarch sightings recorded in the PNW during 2012–24 in each of the four databases used in this study. Columns labeled with the same letter are not significantly different (*p* > 0.5).

During 13 years of observations, the three online sources used in this study (iNaturalist, Western Monarch Milkweed Mapper, and Journey North) recorded 2033 unique sightings of adult monarchs. An additional 1295 unique sightings of adults came from personal communications and social media. Analysis of mean annual sightings showed no significant difference between the online sources (*p* > 0.05) but significantly more from personal communications and social media (ANOVA, *F* = 8.515, df 3, and *p* = 0.04). This was not the case for sightings of immatures, with numbers from the four different sources not significantly different (*p* > 0.5) (Figure 7).

### 3.9. Monarch Sightings by State and Province (2012–24)

**Figure 8:**
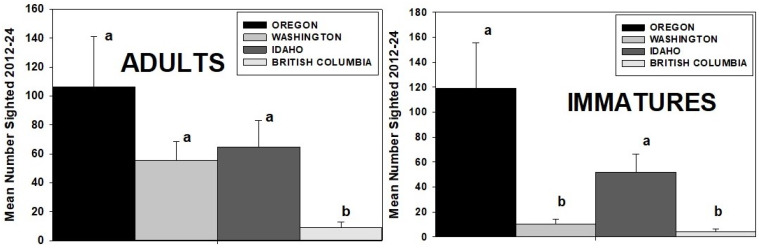
Mean (±SE) number of annual adult and immature monarch sightings in each state and province of the PNW during 2012–24. Columns labeled with a different letter are significantly different (*p* < 0.001).

More adult monarchs were sighted in OR (1381) than in WA (721), ID (843), or BC (120) during the 13-year period of the study. Mean annual sightings for the three states were not significantly different (*p* > 0.05), but significantly fewer sightings were made in BC (ANOVA, *F* = 3.632, df 3, and *p* < 0.001) (Figure 8). Immature monarchs were similarly most frequently sighted in OR (1550), followed by ID (675), WA (137), and BC (51). The number of mean annual sightings was significantly greater in OR and ID than WA and BC (ANOVA, *F* = 7.289, df 3, and *p* < 0.001) (Figure 8).

### 3.10. Adult Monarch Sightings in Western WA and Western BC 2012–24

**Figure 9:**
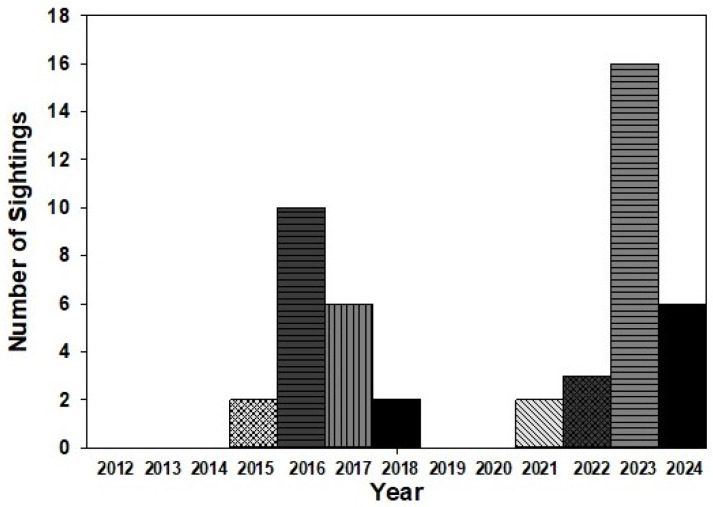
Adult monarch sightings in western Washington and western British Columbia during 2012–24. Sighting data obtained from inaturalist.org, westernmonarchmilkweedmapper.org, journeynorth.org, and from social media and personal communications.

Forty-seven adults, 32 eggs, and five larvae were reported from western WA and western BC during 2012–24. Sightings of adult monarchs occurred mostly in the high abundance years of 2015–17 and 2022–24, with up to 16 sightings annually (Figure 9).

## 4. Discussion

**Figure 10:**
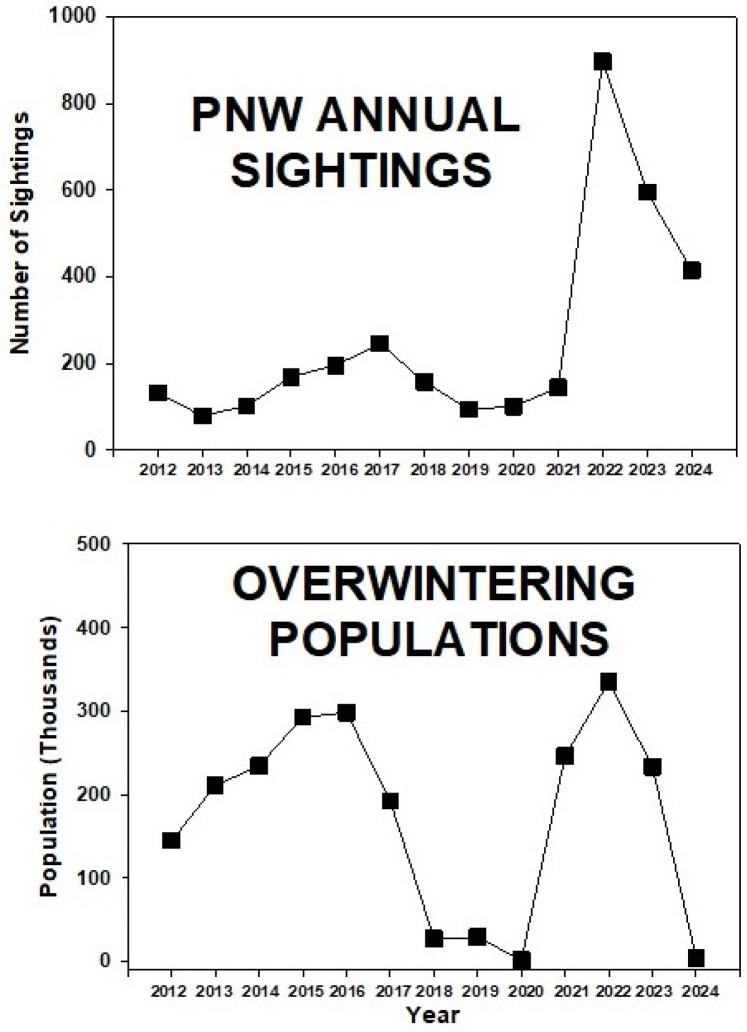
Adult monarch sightings in the PNW and overwintering populations in California during 2012–24. PNW sighting data obtained from inaturalist.org, westernmonarchmilkweedmapper.org, journeynorth.org, and from social media and personal communications. Overwintering population data obtained from https://westernmonarchcount.org/data/ accessed on 31 March 2025.

Although we have a good long-term record of annual fluctuations in the size of overwintering colonies of monarch butterflies in the western US thanks to the annual Thanksgiving Count conducted by hundreds of community scientists [3,15], we lack corresponding data on annual populations of migratory and breeding monarchs in the west. To address this, we used the power of community science by obtaining sighting data from multiple online programs as well as less formal reports from social media and personal communications. By its nature, this kind of data collection suffers from a lack of standardization. However, we believe in this case that the risk of unreliability in some of the data is an acceptable downside given the near-impossibility of obtaining comparable region-wide data by conventional protocols. Our study on monarchs in the PNW over 13 years provides insights into the annual variation in population abundance and phenology, which accords well with the overwintering colony size (Figure 10). Over 13 years, monarch populations in the PNW experienced two three-year periods of higher abundance (2015–17, 2022–24), preceded and separated by two three to four-year periods of lower abundance.

These periods of lower abundance (2012–14, 2018–21) were also years when overwintering numbers were low. Similarly, larger colonies of overwintering monarchs corresponded with larger populations in the PNW (Figure 10). Interestingly, the higher abundance of monarchs during 2015–17 was better represented by spring populations of adults and immatures (Figure 2) than by summer and fall populations (Figure 3 and Figure 4). This suggests, perhaps, that summer populations suffered constraints on population development, possibly caused by natural enemies and/or weather conditions. This may have been an early warning signal for the subsequent population crash at overwintering sites that began in 2018 [1,3].

**Figure 11:**
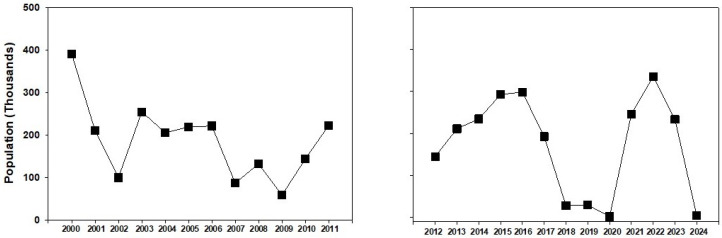
Overwintering monarch populations in California during 2000–2011 and 2012–2024. Data from https://westernmonarchcount.org/data/ accessed on 31 March 2025).

The increased number of sightings during 2022–24, compared to the earlier period of greater abundance during 2015–17, may have partially resulted from greater levels of participation by community scientists rather than a larger population of monarchs. For example, the participation of community scientists on iNaturalist in the US increased from 3 million observations in 2020 to 129 million in 2025 [16]. Overall, our data do not present a picture of continuous decline in monarch abundance during 2012–24 in the PNW. This accords with limited annual community scientist monarch count data in the PNW prior to 2019 that showed a trend of increasing populations [17]. These data do not appear to align with the United States Fish and Wildlife Service’s proposed rule to list the monarch as a threatened species in the United States [4]. Overwintering colonies during 2000–2011 [3] also showed fluctuating 2 to 4-year periods of higher or lower abundance (Figure 11), suggesting that such cyclical fluctuations in monarch abundance may be characteristic of western US populations. The good correspondence between the size of overwintering colonies and the abundance of spring to fall populations during 2012–24 differs from the situation in the eastern US. While overwintering colonies of eastern US monarchs in Mexico have substantially declined [18,19], similar declines in summer-breeding populations have been harder to show [17,20], leading to the hypothesis that mortality during the fall migration has increased [21]. Monarch migration in western North America is much shorter (<1500 km) than migration in eastern North America (3000–4000 km); thus, mortalities related to increased distance and time should be lower, facilitating the observed better correspondence of summer and winter population sizes.

Our annual observations on spring-migrating monarch populations along the Trinity River in Northern CA also showed synchrony with the size of overwintering populations, at least during 2015–16. The larger populations of monarchs in the PNW during 2022–24 were not associated with substantial spring migration along the Trinity River as in earlier high abundance years, perhaps indicating the reduced favorability of this corridor as a migration route. No monarchs were seen along the Trinity River in late spring following the historically low overwintering counts during 2018–2020.

The average first appearance of migrating monarchs in the PNW was about six weeks earlier in OR (mean: 11 May) than in BC (mean: 22 June). First appearance in WA and ID often occurred during the first week of June. Monarchs were reported from OR, WA, ID, and BC in all years of the study. More monarchs were sighted in OR than WA and ID, but only BC had significantly fewer sightings, indicating that this province is the northerly limit of spring migration or that there may be fewer observers in BC. The most northerly sighted monarch (Shuswap, BC) was 200 km north of the international border. The number of adult monarchs seen during spring–fall in the PNW varied substantially from 79 in 2013 to 897 in 2022. The lowest (10) and highest (540) number of sightings of immature monarchs also occurred in 2013 and 2022. Population trends of adult and immature monarchs over 13 years were generally similar regardless of the season of the sighting, except during 2015–17 when summer and fall populations were lower than spring populations. The larger number of monarch sightings during 2022–24 may have been boosted by the increased use of recording sites like iNaturalist. The annual number of wildlife observations on iNaturalist more than doubled between 2018 and 2020 and has continued to grow exponentially [16,22]. Despite this increase in participation, monarch sightings during 2018–20 remained stable and small, adding some credence to the subsequent reported population increase during 2022–24. Our use of multiple recording platforms and sources to collect monarch sighting data minimizes the impact of popularity changes for individual platforms. Surprisingly, significantly more sightings of adult monarchs were collected during the 13-year period in a personal database consisting of personal observations, emails, and the routine monitoring of online monarch-focused social media pages. Using data from multiple sources likely provided a more comprehensive and accurate evaluation than data from a single source.

The presence of monarchs in western WA and western BC has long been considered inconsequential, with most distribution maps showing them absent along the west side of the Cascade Mountains in WA and BC [13,23,24]. In our study, almost 50 monarchs and evidence of breeding were reported from coastal WA and BC, representing 5.6% of our WA and BC sightings. Most sightings were during the years (2015–17, 2022–24) of greatest monarch abundance in the PNW. Sightings were mostly reported in June (51%), likely north-bound spring migrants, with 22.4% in August–September, likely fall migrants. While native milkweeds are uncommon in western areas of the PNW, milkweeds (ornamental and native) are increasingly grown in home gardens in these densely populated, largely urban areas, providing opportunities for monarch breeding. All of the eggs and larvae reported from western WA and western BC were from backyard milkweed patches. Increased cultivation of milkweeds for pollinator conservation [25,26,27] will provide more opportunities for spring-migrating monarchs to encounter and utilize milkweeds for breeding in western WA and western BC, and these areas may host proportionally larger populations in the future.

The direct factors driving the fluctuations in abundance of PNW monarchs observed in this study are unknown but are likely based on adult survival during overwintering and early spring [2] and population growth and survival rates during the multi-generational development of immature stages during spring–fall [28]. A critical period for population growth occurs in early spring in CA when aged and lipid-depleted overwintered females move inland from coastal overwintering sites, seeking scattered and uncommon early season milkweeds [1,29]. The first adult generation that develops on CA milkweeds during March–April migrates north from late April onwards and colonizes the PNW during May and June [24]. The success of spring and summer generations along with the survival of fall migrants determines the extent and size of the subsequent overwintering colonies.

Evaluation of weekly sighting data during June-October over the final three years of the study more precisely showed the phenology and abundance of incoming migrants, and the two summer generations and late summer–fall departure of migrants in the PNW. While the number of incoming migrant sightings in June 2022 was the lowest of the three years, the subsequent locally produced generations produced the greatest number of summer–fall sightings of the three years (and the entire study). In contrast, the high numbers of June migrants and the first local generation of adults in 2024 crashed in mid-August, with low numbers of fall migrants resulting in the second lowest overwintering numbers recorded in California since 1997 [3]. Unlike 2022 and 2023, the development and survival of the second generation in August 2024 appeared to be poor, producing small numbers of adults. Sightings of adults during the second half of August 2024 were reduced by ~75% compared to the previous two years.

Speculation about the possible causes of this apparently widespread and substantial late summer die-off of monarchs in the PNW and other parts of the western US during 2024 has centered on high temperatures [30]. Temperatures above 36 °C under laboratory conditions adversely affect the survival of monarch immatures and adults [31]. A constant temperature of 42 °C for 12 out of 24 h over two days resulted in 80–90% mortality of early and late instar larvae [32]. Field observations in central Washington showed that a heatwave (15 days with daily maxima > 38 °C) had an adverse effect on the survival and persistence of a summer-breeding population of monarchs [12]. While temperatures in the PNW during August 2024 were close to normal [33], the temperatures in July were high above normal (3–4 °C) [34]. From 5 to 21 July, daily maxima ranged from 37.2 to 42.8 °C (mean: 39.4 °C) at the Tri-Cities in south-central WA. This heatwave was widespread, occurring throughout WA, ID, and OR and was comparable to the 15-day heatwave in 2015 (daily maxima > 38 °C) in central WA reported by James [12] that was associated with a 73% decline in a breeding monarch population. The 2015 heatwave coincided with the predominance of eggs and early instar larvae [12]. In 2024, the 17-day July heatwave also likely coincided with the development of eggs and larvae of the second locally produced generation, and the number of ensuing adults was lower and later than in 2022 and 2023, followed by the dramatic crash in numbers of the final generation in mid–late August. Thus, it is possible, if not likely, that the July heatwave in 2024 was at least part of the reason for the late summer decline in the PNW monarch population. The excessive heat was not confined to the PNW; summer 2024 in the western US was one of the warmest recorded [35], with California experiencing the warmest summer on record [36]. The circumstantial evidence presented here suggests that the excessive temperatures experienced in the western US during summer 2024 caused the high mortality of eggs and larvae, which may have been an important driver of the second-lowest overwintering count of monarchs (9119) reported in 2024/25 [3]. The negative impact of heatwaves on butterfly populations is increasingly being recognized [37], and the outsized effect of heat stress on immature stages of butterflies may be an important driver of the declining butterfly populations worldwide under a warming climate [38].

This study illustrates the value and importance of community science observations in helping to understand the phenology and population dynamics of monarch butterflies over a large geographic area and prolonged timeframe. The collection of meaningful data on the occurrence of monarchs over the geography and timescale used in this study would be impossible without community science reporting. The immediacy of community science reporting to sites like iNaturalist, Journey North, Western Monarch Milkweed Mapper, and social media pages allows for the near-real-time monitoring of monarch populations. Given the current and ongoing concern over the status and viability of monarch butterfly populations in North America [39] and the need to act effectively in developing and implementing conservation measures, real-time monitoring of populations is a considerable asset.

## Figures and Tables

**Table 1:** Dates of first and last sightings of monarch butterflies in the Pacific Northwest during 2012–24. Data obtained from inaturalist.org, westernmonarchmilkweedmapper.org, journeynorth.org, and from social media and personal communications.

Year	First Adult				First Egg				First Larva				Last Adult			
State	OR	ID	WA	BC	OR	ID	WA	BC	OR	ID	WA	BC	OR	ID	WA	BC
**2012**	6/1	6/20	5/18	6/24	-	6/20	-	-	6/1	6/30	6/9	-	9/1	8/17	10/11	7/8
**2013**	6/3	6/18	6/6	6/16	-	-	-	-	-	6/29	-	-	9/30	9/18	8/24	8/11
**2014**	5/22	6/6	6/17	6/11	6/16	-	-	-	6/16	6/21	6/26	-	10/5	10/26	5/10	7/29
**2015**	4/10	6/5	5/29	6/7	4/30	-	-	-	6/11	7/11	6/25	-	10/16	9/26	9/28	7/29
**2016**	4/19	5/15	5/31	6/11	5/22	6/12	6/15	7/3	5/28	6/6	6/28		10/19	10/12	9/17	7/27
**2017**	5/3	5/30	6/2	6/11	5/31	7/4	6/30	-	6/15	6/12	6/29	-	10/25	9/21	9/15	7/31
**2018**	4/25	5/23	6/2	7/10	5/17	6/11	-	-	6/14	7/8	-	-	9/30	10/2	10/23	8/13
**2019**	6/1	5/31	5/16	-	8/14	6/12	-	-	8/1	6/12	-	8/6	10/3	10/26	10/26	9/16
**2020**	5/15	6/4	7/14	7/29	7/7	6/10	-		7/8	6/25			9/19	10/5	9/5	7/29
**2021**	6/2	6/11	6/20	7/16	7/28	-	-	-	6/23	7/10	-	-	10/29	10/17	9/26	7/16
**2022**	5/23	5/3	6/8	6/22	6/6	6/23	-	7/5	6/16	6/12	7/7	7/5	10/31	10/25	10/14	9/29
**2023**	4/28	6/5	6/1	6/5	6/6	6/28	6/15	6/15	6/16	6/28	7/10	6/27	11/1	10/22	10/28	10/8
**2024**	4/16	5/29	6/9	6/20	5/24	6/6	-	-	5/31	6/20	7/11	7/3	11/8	10/15	10/26	8/1
**Range**	4/10–6/3	5/3–7/12	5/16–7/14	6/5–7/29	4/30–7/28	6/6–7/4	6/15–6/30	6/15–7/3	5/28–8/1	6/6–7/11	6/15–6/30	6/27–8/6	9/1–11/8	8/17–10/26	8/24–10/28	7/8–10/8
**Mean**	**May 11**	**June 1**	**June 5**	**June 22**	**June 12**	**June 17**	**June 20**	**June 24**	**June 12**	**June 17**	**June 30**	**July 10**	**Oct 14**	**Oct 6**	**Oct 3**	**Aug 9**

## Data Availability

The majority of the data used in this study are available on the community science reporting platforms referenced in the paper (iNaturalist.com, Journeynorth.org, monarchmilkweedmapper.org, Facebook pages: http://facebook.com/MonarchButterfliesInThePacificNorthwest accessed on 31 March 2025, http://facebook.com/groups/813360105993058 accessed on 31 March 2025, http://facebook.com/somonarchs accessed on 31 March 2025, http://facebook.com/groups/2040486716061532 accessed on 31 March 2025, http://facebook.com/groups/1529362950616437 accessed on 31 March 2025, http://facebook.com/groups/626618064195787 accessed on 31 March 2025 http://facebook.com/groups/1453829398271968 accessed on 31 March 2025.
